# Effect of obesity on dolutegravir exposure in Black Southern African adults living with HIV

**DOI:** 10.4102/sajhivmed.v23i1.1452

**Published:** 2022-12-13

**Authors:** Enkosi Mondleki, Clifford G. Banda, Nomathemba C. Chandiwana, Simiso Sokhela, Lubbe Wiesner, Francois Venter, Gary Maartens, Phumla Z. Sinxadi

**Affiliations:** 1 Division of Clinical Pharmacology, Department of Medicine, Faculty of Health Sciences, University of Cape Town, Cape Town, South Africa; 2 Malawi-Liverpool-Wellcome Trust Clinical Research Programme, Blantyre, Malawi; 3 Ezintsha, University of the Witwatersrand, Johannesburg, South Africa; 4 Wellcome Centre for Infectious Diseases Research in Africa, Institute of Infectious Disease and Molecular Medicine, University of Cape Town, Cape Town, South Africa

**Keywords:** pharmacokinetics, dolutegravir, obesity, South Africa, antiretroviral treatment optimisation, HIV

## Abstract

**Background:**

Dolutegravir, a component of the preferred first-line antiretroviral therapy regimen, has been associated with increased weight gain. South Africa has a high prevalence of obesity, especially among women. Understanding dolutegravir exposure in patients with obesity is important for dose optimisation.

**Objectives:**

We compared the pharmacokinetic parameters of dolutegravir in Southern African adults living with HIV with and without obesity.

**Method:**

Blood samples were collected at various time points over a 24 h-period for dolutegravir assays. Non-compartmental analysis was conducted and geometric mean ratios (GMRs), with 90% confidence intervals (CIs), were generated to compare dolutegravir pharmacokinetic parameters between the groups. Regression analyses to assess predictors of dolutegravir exposure were done.

**Results:**

Forty participants were enrolled, 26 were women and 10 had obesity. Dolutegravir area under the concentration-time curve to 24-h and the maximum concentrations were not statistically significantly lower in participants with obesity: GMR 0.91 (90% CI: 0.71–1.16) and GMR 0.86 (90% CI: 0.68–1.07), respectively. In a multivariate linear regression analysis adjusting for age, gender, body mass index, creatinine clearance and randomisation arm (tenofovir alafenamide or tenofovir disoproxil fumarate), a unit increase in body mass index was associated with 1.2% lower dolutegravir area under the concentration-time curve to 24-h (*P* = 0.035).

**Conclusion:**

Dolutegravir exposure was marginally lower in participants with obesity, but this is not clinically significant. Our findings suggest that there is no need to dose adjust dolutegravir in people with obesity.

## Introduction

Antiretroviral therapy (ART) has reduced morbidity and mortality in patients living with HIV.^1^ Antiretroviral therapy regimens with durable efficacy, better tolerability and long-term safety are now preferred.^2^ In all current HIV treatment guidelines, second-generation integrase strand transfer inhibitors, such as dolutegravir, are included in first-line ART regimens owing to their excellent tolerability and high resistance barrier.^3,4^

Although weight gain can be regarded as an appropriate ‘return-to-health’ phenomenon after initiating ART with any class, excessive weight gain can lead to treatment-emergent obesity.^5^ In pooled analyses of eight randomised clinical trials with more than 5000 participants with more than 10 000 person years of follow-up, more weight gain was associated with the use of integrase strand transfer inhibitors in ART-naïve people living with HIV (PLWH) than with other classes of antiretrovirals.^5,6^ Over 96 weeks after initiating ART, the proportion of participants who were overweight or obese increased from 31.4% to 34.7% and from 16.3% to 21.4%, respectively.^6^ In sub-Saharan Africa, two randomised controlled trials conducted in ART-naïve PLWH compared dolutegravir with efavirenz (standard-dose efavirenz in ADVANCE^7^ and low-dose efavirenz in NAMSAL^8^) – the ADVANCE trial dolutegravir was combined with emtricitabine and tenofovir disoproxil fumarate (TDF) or tenofovir alafenamide (TAF). Both of these studies reported more weight gain and treatment-emergent obesity in participants treated with dolutegravir compared with efavirenz. In the NAMSAL trial, treatment-emergent obesity at 48 weeks was 12% in the dolutegravir arm and 5% in the low-dose efavirenz arm.^8^ In the ADVANCE trial, treatment-emergent-obesity at 96 weeks was 19% for the dolutegravir-TAF arm, 8% for dolutegravir-TDF, and 4% for efavirenz-TDF.^7^ The weight gain and obesity were more marked in women. The OPERA cohort of PLWH, reported that switching from TDF to TAF was associated with weight gain.^9,10,11^

Obesity, is a common outcome of all modern ART regimens, especially among Black women.^6,12,13^ In South Africa, there are 7.8 million PLWH, with 230 000 new HIV infections reported in 2020.^14^ South Africa also has a high level of pre-existing obesity: 68% of women and 31% of men were overweight or obese in a 2016 survey.^15^ Obesity affects several physiological processes relevant to drug exposure (e.g. gut permeability, gastric emptying, cardiac output, liver and renal function).^16^ It is important to determine if drug exposure is sub-optimal in obese individuals as they are usually excluded in drug development studies that inform dosing.^17,18^

It has been postulated that dolutegravir could cause weight gain by off-target effects through inhibition of the melanocortin-4 receptor pathway, affecting appetite and energy balance.^19,20^ However, *in vitro* studies have shown that the concentrations needed for the direct inhibition of the melanocortin-4 receptor that would explain clinically important weight gain are much higher than those achieved with the currently recommended daily dose of 50 mg.^21^ In a sub-study of ADVANCE, our group has recently shown that weight gain differences between dolutegravir and efavirenz are driven by impaired weight gain in participants who are genetically slow metabolisers of efavirenz^22^ – this finding suggests that dolutegravir is not causing weight gain but that efavirenz is impairing weight gain in slow metabolisers who have high efavirenz concentrations. The reason for the contributory effect of TAF on weight gain is still unclear and may reflect weight loss effects of TDF.^12,23^

As marked weight gain is increasingly reported in patients treated with dolutegravir, especially when co-administered with TAF,^5,7^ understanding the effects of obesity on dolutegravir exposure is important for dose optimisation to ensure the efficacy and safety dolutegravir in patients with obesity.^17,24,25^

Dolutegravir is a highly protein-bound, non-lipophilic, slightly water soluble drug, with a modest apparent volume of distribution.^4,26^ Pharmacokinetic studies in obesity show that the behaviour of molecules with weak or moderate lipophilicity is generally predictable, as these drugs are distributed mainly in lean tissues.^27^ However, some of these drugs are partly distributed in adipose tissues, and their dosage should be based on ideal body weight plus a percentage of the patient’s excess bodyweight.^27^ Data comparing dolutegravir exposure in the patients with obesity are lacking. We hypothesised that dolutegravir exposure would be lower in participants with obesity compared to those without, due to the pharmacokinetic changes observed in obesity. We compared the pharmacokinetic parameters of dolutegravir administered in participants with and without obesity in Southern African PLWH enrolled in the ADVANCE randomised clinical trial. We also explored covariates associated with overall dolutegravir exposure.

## Research methods and design

### Study population and study design

The ADVANCE study (NCT03122262) was a Phase III clinical trial conducted in South Africa, which randomised 1053 ART-naïve participants to one of three treatment arms: (1) dolutegravir, TAF and emtricitabine; (2) dolutegravir, TDF and emtricitabine; or (3) efavirenz, TDF and emtricitabine.^5^ The present pharmacokinetic sub-study included participants from the ADVANCE study who were older than 18 years of age, weighed 40 kg or more, were randomised to the dolutegravir arms, and consented to the intensive pharmacokinetic sub-study.

All participants included had already completed at least 96 weeks of therapy. We excluded those who missed any ART doses within three days before the pharmacokinetic sampling, smokers, and participants who needed concomitant medications with a potential for drug-drug interactions with dolutegravir. We used the World Health Organization definition to categorise participants into two groups: those with obesity (≥ 30 kg/m^2^) and those without obesity (< 30 kg/m^2^).^28^

### Pharmacokinetic sampling and analysis

Enrolled participants had a standardised meal prior to observed oral administration of the study medication. Blood sampling was done at 0 (pre-dose), 1, 2, 4, 6, 8 and 24-h post dosing. An intravenous cannula was inserted and remained in situ for serial sampling up to 8 h. At each time point, 4 mL of venous blood was collected in an ethylenediaminetetraacetic acid tube, centrifuged, plasma pipetted, and stored at −80 °C until analysis.

Dolutegravir was quantified with a validated assay developed at the Division of Clinical Pharmacology, University of Cape Town. Samples were processed with a liquid-liquid extraction method using dolutegravir-d4 as an internal standard, followed by high performance liquid chromatography with tandem mass spectrometry detection using an AB Sciex API 4000 triple quadrupole mass spectrometer (AB Sciex™, Darmstadt, Germany). Analyte and internal standards were monitored at mass transitions of the protonated precursor ions (mass to charge ratio 420:1 and 424:2) to the product ions (mass to charge ratio 277:2 and 279:1), respectively. The calibration curve fitted a quadratic regression over the range 0.030 µg/mL to 10.0 µg/mL. Combined accuracy and precision statistics of quality control samples during validation were between 103.5% and 106.0%, and 4.6% and 6.1%, respectively. The laboratory participated in the Clinical Pharmacology Quality Assurance external quality control programme under a contract with the Division of AIDS of the National Institute of Allergy and Infectious Diseases, through which this assay was approved.

### Statistical analysis

We conducted secondary analyses of 40 study participants enrolled in the intensive pharmacokinetic sampling sub-study (20 in each of the dolutegravir-based arms) and categorised them into participants with or without obesity.

Baseline characteristics were described using medians (interquartile ranges) for non-parametric continuous variables and proportions (%) for categorical variables.

Using non-compartmental analysis, employing the trapezoidal rule with cubic splines, the following pharmacokinetic parameters were estimated for dolutegravir: the area under the concentration-time curve to the last measurable time point at 24 h post dosing (AUC_0–24h_), terminal elimination half-life (t_1/2_), maximum concentration (C_max_) and time to C_max_ (T_max_). The apparent clearance of dolutegravir was calculated using the equation dose/AUC_0–24h_, while the trough concentrations were estimated from the sample collected just before the next dose. Pharmacokinetic data were log-transformed to calculate the geometric mean ratio (GMR) of the pharmacokinetic parameters of dolutegravir comparing participants with obesity to those without with 90% confidence intervals (CI) evaluated using paired *t*-tests and back-transformed to absolute ng/mL concentrations. Changes in pharmacokinetic parameters between the two arms were considered statistically significant when the 90% CI of the GMR did not cross the value of one. Multivariate linear regression was used to explore and determine covariates associated with overall drug exposure (AUC_0–24_). The covariates explored were age, gender, body mass index (BMI), creatinine clearance and the ART regimen group (TAF vs TDF). A *P*-value of < 0.05 was considered as significant. There was no correction for multiple testing.

All the analyses were conducted in Stata^®^ (version 16.0, StataCorp LLC, College Station, Texas, United States [US]).

### Ethical considerations

This sub-study was approved by the University of the Witwatersrand Human Research Ethics Committee (Wits HREC 160606B) and the University of Cape Town Human Research Ethics Committee (HREC REF: 224/2021). All participants provided additional written informed consent to participate in the pharmacokinetic sub-study. Participant samples were labelled with coded identifiers to protect confidentiality and databases were password-protected. All procedures performed in studies involving human participants were in accordance with the ethical standards of the institutional and/or national research committee and with the 1964 Helsinki Declaration and its later amendments.

## Results

**FIGURE 1:**
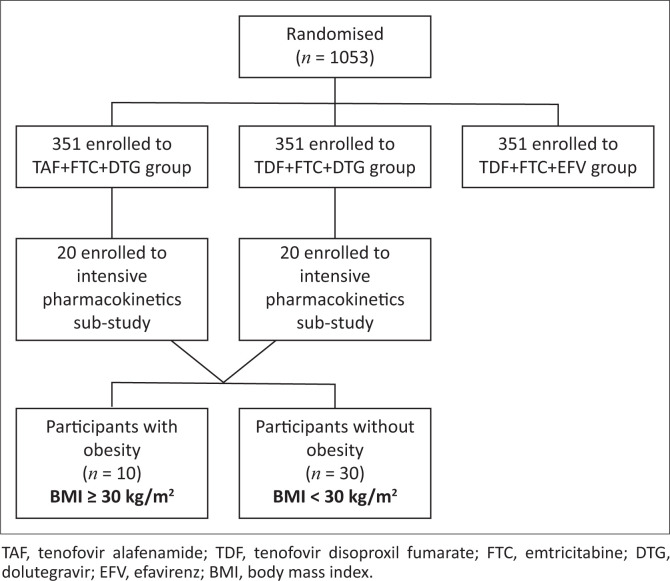
Participant flow chart.

Forty participants were enrolled into the intensive pharmacokinetic sub-study. The participant flow chart is shown in Figure 1. Ten participants were classified as participants with obesity, and their baseline characteristics are summarised in Table 1.

**TABLE 1:** Baseline characteristics of study participants included in analysis (*N* = 40).

Variables	All				Participants with obesity (*n* = 10)				Participants without obesity (*n* = 30)			
	*N*	%	Median	IQR	*N*	%	Median	IQR	*N*	%	Median	IQR
Age in years	-	-	32	29 to 37	-	-	35	31 to 44	-	-	31	28 to 36
**Gender**												
Female	26	65	-	-	8	80	-	-	18	60	-	-
Weight (kg)	-	-	73.7	67.3 to 85.6	-	-	90.6	86.0 to 95.3	-	-	69.1	62.1 to 75.5
Height (cm)	-	-	166.5	160 to 173.5	-	-	165	159.0 to 172.0	-	-	167	161 to 175
Body mass index (kg/m^2^)	-	-	27.1	23.4. to 30.19	-	-	32.8	31.7.8 to 34.4	-	-	25.3	22.6 to 27.9
**Antiretroviral therapy regimen**												
Tenofovir alafenamide/emtricitabine/dolutegravir	20	100	-	-	5	50	-	-	15	50	-	-
Tenofovir disoproxil fumarate/emtricitabine/dolutegravir	20	100	-	-	5	50	-	-	15	50	-	-
Baseline creatinine (µmol/L)	-	-	70	56.0 to 76.0	-	-	73	56.0 to 75.0	-	-	67.0	56.0 to 77.0
Baseline creatinine clearance (mL/ min)	-	-	130.5	104.5 to 148.5	-	-	135.6	126.3 to 181.0	-	-	119.5	103.8 to 145.6

Note: Medians and interquartile ranges were used to describe continuous variables. Proportions (*n*, [%]) were used to describe categorical variables.

### Pharmacokinetic profile of dolutegravir in participants with versus without obesity

**FIGURE 2:**
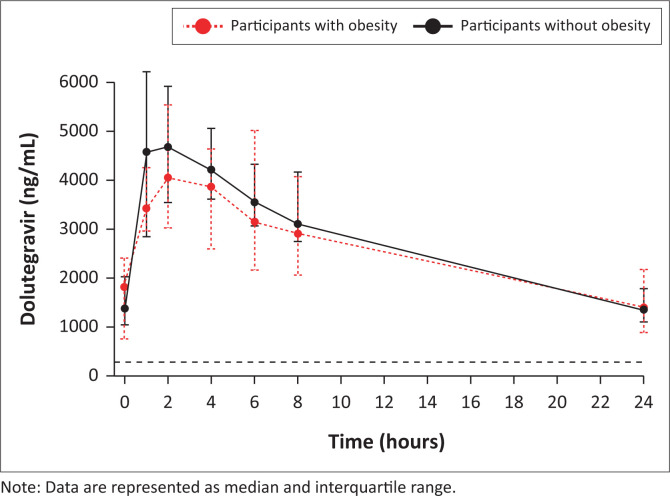
Median concentration-time of dolutegravir administered in participants with obesity (red line plot, *n* = 10) and participants without obesity (black line plot, *n* = 30). The black dashed horizontal line represents the putative minimum effective dolutegravir concentration of 300 ng/mL.

Pharmacokinetic parameters of dolutegravir when administered in participants with and without obesity are summarised in Table 2. Numerical reductions in the AUC_0–24h_ (9%) and C_max_ (14%) were observed in the participants with obesity, however, these were not statistically significant. The time to C_max_ was significantly prolonged (62%) in the participants with obesity. There were no differences in apparent dolutegravir clearance between the two groups. The median (interquartile range) concentration-time profiles of dolutegravir in the participants with and without obesity are shown in Figure 2. In both groups, dolutegravir trough concentrations were above the putative minimum effective concentration of 300 ng/mL.^29,30^

**TABLE 2:** Dolutegravir exposure profile in participants with obesity (*n* = 10) compared with participants without obesity (*n* = 30).

Pharmacokinetic parameter	DTG in participants with obesity (Group 1)		DTG in participants without obesity (Group 2)		Group 2/Group 1		*P*
	Geometric mean (GM)	90% CI	Geometric mean (GM)	90% CI	GM ratio	90% CI	
AUC_0–24h_ (ng.h/mL)	62 502	50 178 to 77 852	68 491	62 153 to 75 475	0.91	0.71 to 1.16	0.529
C_max_ (ng/mL)	4 268	3 502 to 5 201	4 985	4 504 to 5 518	0.86	0.68 to 1.07	0.251
C_24_ (ng/mL)	1 433	1 073 to 1 912	1 444	1 263 to 1 650	0.99	0.72 to 1.37	0.968
T_max_ (h)	2.4	1.8 to 3.3	1.5	1.3 to 1.7	**1.62**	**1.15 to 2.27**	**0.023**
t_1/2_ (h)	16.4	13.2 to 20.3	14.7	13.1 to 16.5	1.11	0.87 to 1.42	0.470
CL/F (litres/h)	0.80	0.64 to 1.00	0.73	0.66 to 0.80	1.10	0.86 to 1.40	0.529

Note: Comparisons done with independent t-test, bold represents statistical significance.

CI, confidence interval; DTG, dolutegravir; C_max_, maximum concentration; C_24_, trough concentrations; T_max_, time to maximum concentration; t_1/2_, terminal elimination half-life; CL/F, oral clearance; AUC_0–24_, area under the concentration-time curve to the last measurable time point at 24 h post dosing.

### Predictors of overall dolutegravir exposure

In a multivariate linear regression analysis to investigate covariates associated with dolutegravir AUC_0–24h_ in the whole group, a unit increase in BMI was associated with 1.2% lower dolutegravir exposure (beta coefficient = −1838.66, 95% CI: −3540.85 to −136.46, *P* = 0.035) (Table 3). Other covariates tested (age, gender, creatinine clearance, and treatment groups [TDF or TAF]) were not associated with dolutegravir exposure (Table 3).

**TABLE 3:** Association between dolutegravir exposure (area under the concentration-time curve to the last measurable time point at 24 h post dosing) and various predictive covariates.

Variable	Unadjusted			Adjusted		
	Beta coefficient	95% CI	*P*	Beta coefficient	95% CI	*P*
BMI	−1 343.60	−2 889.39 to 202.18	0.087	**−1 838.66**	**−3 540.85 to −136.46**	**0.035**
ART treatment group (TDF)	−5 458.93	−20 341.69 to 9 423.84	0.462	−4 835.94	−19 222.67 to 9 550.78	0.499
Age	−652.87	−1 632.07 to 326.32	0.185	83.94	−1 055.50 to 1 223.38	0.882
Gender (male)	−12 537.39	−27 702.57 to 2 627.78	0.102	−14 975.44	−39 693.31 to 9 742.42	0.227
Creatinine clearance	−373.78	−787.00 to 39.46	0.075	−108.51	−700.92 to 483.90	0.712

Note: Bold represents statistical significance.

CI, confidence interval; BMI, body mass index; ART, antiretroviral treatment; TDF, tenofovir disoproxil fumarate.

## Discussion

We investigated the effects of obesity on dolutegravir pharmacokinetics among participants enrolled into the intensive pharmacokinetic sampling sub-study of the ADVANCE study. We investigated predictors of dolutegravir exposure in the whole group using multivariate regression analyses adjusting for age, gender, BMI, creatinine clearance, and tenofovir prodrug: only BMI was independently associated with higher dolutegravir AUC_0–24h_. In the group of participants with obesity, we found that AUC_0–24h_ and C_max_ were marginally lower. However, this was not statistically significant. We also observed that the time to C_max_ was significantly prolonged in this group. However, these observed minor differences in pharmacokinetic parameters between the groups are not clinically significant.

Obesity is associated with various physiological changes that can affect drug pharmacokinetics. These include changes in plasma proteins, drug metabolising enzymes, drug transporters and blood flow.^16^ In our study, we observed a marginal, non-significant, decrease in overall dolutegravir exposure in the group of participants with obesity. The time to C_max_ was significantly prolonged in this group, which was a surprising finding as drug absorption is not generally affected by obesity.^16,25,31^

Similar observations were made in the Swiss HIV cohort study using a physiologically based pharmacokinetic modelling, where obesity was predicted to reduce dolutegravir C_max_ and AUC by 13% and 3%, respectively.^32^ The observed marginal reduction in dolutegravir exposure in our study is not clinically significant as all participants had concentrations above the putative minimum effective concentration of 300 ng/mL.^29,30^ We investigated predictors of dolutegravir and found a unit increase in BMI that was associated with a significantly lower dolutegravir AUC_0–24h_. However, this small difference is not clinically significant.

Our study has limitations. First, this was a post hoc analysis, and we did not do formal sample size calculations. The sample size of 10 participants with, and 30 without, obesity had limited power to detect small differences in overall dolutegravir exposure. The post hoc power estimation showed that a sample size that included 10 participants with obesity and 30 without would provide 80% power if the relative difference in AUC between these groups was 30%. Second, we classified our participants into those with versus without obesity; however, the group of participants without obesity also included 17 participants who were overweight (BMI: > 25 kg/m^2^ to < 30 kg/m^2^). This may have underestimated the impact of obesity on dolutegravir exposure. In a sensitivity analysis comparing the 10 participants with obesity with 13 adults with a healthy weight (BMI: ≤ 25 kg/m^2^), the dolutegravir pharmacokinetic profile was similar to that seen when overweight participants were included (data not shown). Third, all our participants were Africans; our findings may, therefore, not be generalisable to other populations.

## Conclusion

Dolutegravir exposure was marginally lower in participants with obesity, but this is not clinically significant given that trough concentrations were above a putative minimum effective concentration. Our findings suggest that there is no need to dose adjust dolutegravir in patients with obesity. However, future research studies with larger sample size are warranted.

## References

[1] Ray AS, Fordyce MW, Hitchcock MJ. Tenofovir alafenamide: A novel prodrug of tenofovir for the treatment of Human Immunodeficiency Virus. Antiviral Res. 2016;125:63–70. 10.1016/j.antiviral.2015.11.009 26640223

[2] Ruane PJ, DeJesus E, Berger D, et al. Antiviral activity, safety, and pharmacokinetics/pharmacodynamics of tenofovir alafenamide as 10-day monotherapy in HIV-1-positive adults. J Acquir Immune Defic Syndr. 2013;63(4):449–455. 10.1097/QAI.0b013e3182965d45 23807155

[3] NDoH. 2019 ART clinical guidelines for the management of HIV in adults, pregnancy, adolescents, children, infants and neonates [homepage on the Internet]. National Department of Health; 2019 [cited 2022 Feb 14]. Available from: https://www.health.gov.za/wp-content/uploads/2020/11/2019-art-guideline.pdf

[4] Walmsley SL, Antela A, Clumeck N, et al. Dolutegravir plus abacavir-lamivudine for the treatment of HIV-1 infection. N Engl J Med. 2013;369(19):1807–1818. 10.1056/NEJMoa1215541 24195548

[5] Eckard AR, McComsey GA. Weight gain and integrase inhibitors. Curr Opin Infect Dis. 2020;33(1):10–19. 10.1097/QCO.0000000000000616 31789693 PMC7433018

[6] Sax PE, Erlandson KM, Lake JE, et al. Weight gain following initiation of antiretroviral therapy: Risk factors in randomized comparative clinical trials. Clin Infect Dis. 2020;71(6):1379–1389. 10.1093/cid/ciz999 31606734 PMC7486849

[7] Venter WDF, Moorhouse M, Sokhela S, et al. Dolutegravir plus two different prodrugs of tenofovir to treat HIV. N Engl J Med. 2019;381(9):803–815. 10.1056/NEJMoa1902824 31339677

[8] Calmy A, Tovar Sanchez T, Kouanfack C, et al. Dolutegravir-based and low-dose efavirenz-based regimen for the initial treatment of HIV-1 infection (NAMSAL): Week 96 results from a two-group, multicentre, randomised, open label, phase 3 non-inferiority trial in Cameroon. Lancet HIV. 2020;7(10):e677–e687. 33010241 10.1016/S2352-3018(20)30238-1

[9] Mallon PW, Brunet L, Hsu RK, et al. Weight gain before and after switch from TDF to TAF in a U.S. cohort study. J Int AIDS Soc. 2021;24(4):e25702. 10.1002/jia2.25702 33838004 PMC8035674

[10] Leonard MA, Cindi Z, Bradford Y, et al. Efavirenz pharmacogenetics and weight gain following switch to integrase inhibitor-containing regimens. Clin Infect Dis. 2021;73(7):e2153–e2163. 10.1093/cid/ciaa1219 32829410 PMC8492125

[11] Turkova A, White E, Mujuru HA, et al. Dolutegravir as first- or second-line treatment for HIV-1 infection in children. N Engl J Med. 2021;385(27):2531–2543. 10.1056/NEJMoa2108793 34965338 PMC7614690

[12] Shah S, Hindley L, Hill A. Are new antiretroviral treatments increasing the risk of weight gain? Drugs. 2021;81(3):299–315. 10.1007/s40265-020-01457-y 33400239

[13] Mabaso M, Makola L, Naidoo I, Mlangeni LL, Jooste S, Simbayi L. HIV prevalence in South Africa through gender and racial lenses: Results from the 2012 population-based national household survey. Int J Equity Health. 2019;18(1):167. 10.1186/s12939-019-1055-6 31666077 PMC6821038

[14] UNAIDS. UNAIDS data 2021 [homepage on the Internet]. UNAIDS; 2021 [cited 2022 Feb 14]. Available from: https://www.unaids.org/sites/default/files/media_asset/JC3032_AIDS_Data_book_2021_En.pdf

[15] NDoH, StatsSA, SAMRC, ICF. South Africa demographic and health survey 2016 [homepage on the Internet]. National Department of Health; 2019 [cited 2022 Feb 14]. Available from: https://dhsprogram.com/pubs/pdf/FR337/FR337.pdf

[16] Jain R, Chung SM, Jain L, et al. Implications of obesity for drug therapy: Limitations and challenges. Clin Pharmacol Ther. 2011;90(1):77–89. 10.1038/clpt.2011.104 21633345

[17] CMHP. Reflection paper on investigation of pharmacokinetics and pharmacodynamics in the obese population [homepage on the Internet]. European Medicines Agency; 2018 [cited 2022 Feb 14]. Available from: https://www.ema.europa.eu/en/documents/scientific-guideline/reflection-paper-investigation-pharmacokinetics-pharmacodynamics-obese-population_en.pdf

[18] Madelain V, Le MP, Champenois K, et al. Impact of obesity on antiretroviral pharmacokinetics and immuno-virological response in HIV-infected patients: A case-control study. J Antimicrob Chemother. 2017;72(4):1137–1146. 10.1093/jac/dkw527 28065890 PMC5517629

[19] Griesel R, Kawuma AN, Wasmann R, et al. Concentration-response relationships of dolutegravir and efavirenz with weight change after starting antiretroviral therapy. Br J Clin Pharmacol. 2022;88(3):883–893. 10.1111/bcp.15177 34954840 PMC7612404

[20] Adan RA, Tiesjema B, Hillebrand JJ, La Fleur SE, Kas MJ, De Krom M. The MC4 receptor and control of appetite. Br J Pharmacol. 2006;149(7):815–827. 10.1038/sj.bjp.0706929 17043670 PMC2014686

[21] McMahon C, Trevaskis JL, Carter C, et al. Lack of an association between clinical INSTI-related body weight gain and direct interference with MC4 receptor (MC4R), a key central regulator of body weight. PLoS One. 2020;15(2):e0229617. 10.1371/journal.pone.0229617 32109250 PMC7048285

[22] Griesel R, Maartens G, Chirehwa M, et al. CYP2B6 genotype and weight gain differences between dolutegravir and efavirenz. Clin Infect Dis. 2021;73(11):e3902–e3909. 10.1093/cid/ciaa1073 32960272 PMC8653639

[23] Lake JE, Trevillyan J. Impact of Integrase inhibitors and tenofovir alafenamide on weight gain in people with HIV. Curr Opin HIV AIDS. 2021;16(3):148–151. 10.1097/COH.0000000000000680 33797433

[24] Cho S-J, Yoon I-S, Kim D-D. Obesity-related physiological changes and their pharmacokinetic consequences. J Pharm Investig. 2013;43:161–169. 10.1007/s40005-013-0073-4

[25] Gounden R, Blockman M. Dosing in an obese patient: Clinical pharmacology. CME. 2006;24:399–400.

[26] Healthcare V. Tivicay [homepage on the Internet]. 2013 [cited 2022 Oct 29]. Available from: https://www.ema.europa.eu/en/documents/product-information/tivicay-epar-product-information_en.pdf

[27] Cheymol G. Effects of obesity on pharmacokinetics implications for drug therapy. Clin Pharmacokinet. 2000;39(3):215–231. 10.2165/00003088-200039030-00004 11020136

[28] WHO. Obesity: Preventing and managing the global epidemic. Report of a WHO consultation on obesity, 1997 Jun 03–05; Geneva. WHO; 1997 [cited 2022 Feb 14]. Available from: https://apps.who.int/iris/handle/10665/63854 11234459

[29] Walimbwa SI, Lamorde M, Waitt C, et al. Drug interactions between dolutegravir and artemether-lumefantrine or artesunate-amodiaquine. Antimicrob Agents Chemother. 2019;63(2):e01310–e01318. 10.1128/AAC.01310-18 30420479 PMC6355558

[30] Dooley KE, Savic R, Gupte A, et al. Once-weekly rifapentine and isoniazid for tuberculosis prevention in patients with HIV taking dolutegravir-based antiretroviral therapy: A phase 1/2 trial. Lancet HIV. 2020;7(6):e401–e409. 10.1016/S2352-3018(20)30032-1 32240629

[31] Smit C, De Hoogd S, Bruggemann RJM, Knibbe CAJ. Obesity and drug pharmacology: A review of the influence of obesity on pharmacokinetic and pharmacodynamic parameters. Expert Opin Drug Metab Toxicol. 2018;14(3):275–285. 10.1080/17425255.2018.1440287 29431542

[32] Berton M, Bettonte S, Decosterd L, et al. Pharmacokinetics of dolutegravir and bictegravir in obese people living with HIV. Denver, CO: Conference on retroviruses and opportunistic infections (CROI); 2022 Feb 12–16.

